# A Monte Carlo test of linkage disequilibrium for single nucleotide polymorphisms

**DOI:** 10.1186/1756-0500-4-124

**Published:** 2011-04-14

**Authors:** Hongyan Xu, Varghese George

**Affiliations:** 1 Department of Biostatistics, Georgia Health Sciences University, Augusta,GA, USA

## Abstract

**Background:**

Genetic association studies, especially genome-wide studies, make use of linkage disequilibrium(LD) information between single nucleotide polymorphisms (SNPs). LD is also used for studying genome structure and has been valuable for evolutionary studies. The strength of LD is commonly measured by *r*^2^, a statistic closely related to the Pearson's *χ*^2 ^statistic. However, the computation and testing of linkage disequilibrium using *r*^2 ^requires known haplotype counts of the SNP pair, which can be a problem for most population-based studies where the haplotype phase is unknown. Most statistical genetic packages use likelihood-based methods to infer haplotypes. However, the variability of haplotype estimation needs to be accounted for in the test for linkage disequilibrium.

**Findings:**

We develop a Monte Carlo based test for LD based on the null distribution of the *r*^2 ^statistic. Our test is based on *r*^2 ^and can be reported together with *r*^2^. Simulation studies show that it offers slightly better power than existing methods.

**Conclusions:**

Our approach provides an alternative test for LD and has been implemented as a R program for ease of use. It also provides a general framework to account for other haplotype inference methods in LD testing.

## Background

Genetic association studies, especially large-scale genome-wide association studies have become very popular in recent years due to the rapid advancement of genotyping technologies and the completion of the Human Genome Project [1,2]. More than 400 susceptibility regions have been identified through genome-wide association approach. This approach relies on the linkage disequilibrium information between genetic markers, mostly single-nucleotide polymorphisms (SNPs), hence been termed linkage disequilibrium mapping. Linkage disequilibrium (LD) refers to the nonrandom association of alleles at different loci on the same haplotype. The underlying assumption of genetic association studies is that there are some disease causing loci in the genome, and if the SNPs under investigation (i.e. markers) and the disease-causing loci are in close proximity, the marker alleles will be associated with the alleles at the disease-causing loci. In other words, those markers are in LD with the disease causing loci if they are in close proximity. Since markers in high LD are highly correlated, testing the significance of LD between alleles of markers is also useful in finding LD blocks and tag-SNPs. This could reduce the number of markers required in genome-wide studies. In addition to gene mapping, LD information also proves to be useful in evolutionary studies of gene dynamics, tracing human origin and history, and studies of genome structure and forensic science.

Consider two bi-allelic SNPs, marker A and marker B. The two alleles at marker A are denoted as *A*_1 _and *A*_2 _with frequencies *p*_1 _and *p*_2_, respectively, and the two alleles at marker B are denoted as *B*_1 _and *B*_2 _with frequencies *q*_1 _and *q*_2 _respectively. The non-random association of the alleles at the two loci can be measured as the difference between the haplotype frequency of *A*_1_*B*_1 _in the population and the expected frequency under the null hypothesis of independence i.e., , where  is the frequency of haplotype *A*_1_*B*_1_. If we replace the population haplotype frequency of *A*_1_*B*_1_, , by the observed frequency,  in the sample, we get an estimator of *δ*, given by . The statistic *D *depends on marker allele frequency, which makes it harder to compare across different markers and populations. As a result, many measures have been proposed to standardize *D*. Two such common measures of LD are *D' *[3] and r^2 ^[4]. *D' *is bounded between 0 and 1. The bound of *r*^2 ^depends on allele frequency and is given in [4].

The first measure is *D' *= *D*/*D_max_*, where *D_max _*is the upper bound on D, given by,

The other popular measure of LD, denoted *r*^2^, is the correlation of alleles at the two bi-allelic loci, defined as,(1)

In general, *r*^2 ^is used to measure the statistical association between marker pairs and is related to the power of LD mapping. In a case-control study, if *r*^2 ^is the level of LD between a marker and a causative polymorphism and the sample size required to detect the association of the disease with the causative polymorphism is *n*, then the sample size required to detect the association of the disease with the marker at the same power level is approximately equal to *n*/*r*^2 ^[5-7]. Because of this convenient relationship, *r*^2 ^is used extensively in association mapping as a measure of LD.

*r*^2 ^is also closely related to the Pearson's *χ*^2 ^statistic for testing the association of alleles at two loci. For two SNP markers A and B, each having 2 alleles, we can construct a 2 × 2 contingency table containing the haplotype counts. We could then compute the Person's *χ*^2 ^statistic with 1 degree of freedom based on the contingency table. The LD measure *r*^2 ^can then be written as,(2)

where *N *is the number of chromosomes in the sample, or twice the number of individuals for humans. *Nr*^2 ^is then compared to a -distribution as a test of LD. This works fine when the haplotypes can be directly observed. However, problems arises when we use this approach in the analysis of population-based data, where haplotypes are usually not observed so the cell counts of the contingency table are not known. As a result, an estimation procedure, such as maximum-likelihood approach, has to be used to estimate the haplotype counts. This introduces additional variability and in turn the test statistic *Nr*^2 ^will not follow a -distribution. For example, in the R package, "genetics", the estimated haplotype counts are used to compute *Nr*^2^, which is then compared to a -distribution as a test of LD, although there is a warning in the documentation noting that this may not be a valid test.

An approach that allows for unknown haplotypes in testing LD has been proposed by Weir [8], based on a composite LD measure. This approach has been extended to markers with multiple alleles by Schaid [9] and Zaykin *et al*. [10]. A test of LD based on the common measure *r*^2 ^has been developed on the asymptotic distribution derived from the *δ*-method [11]. In this report, we first show that the additional variability from haplotype estimation has to be accounted for in a test of LD when haplotype frequencies are not available. We then propose a test that accounts for this variability and present its properties in terms of type I error rate and power. Finally we compare the our test with that based on the composite LD and the test based on the asymptotic distribution.

## Methods

### Effects of haplotype estimation

As mentioned above, in most population-based studies where haplotypes are not directly observable, the haplotype counts have to be estimated. Most of the estimation procedures are based on maximum-likelihood approach as implemented in the R package "genetics", which is freely availably from the CRAN website http://www.cran.org. This estimation procedure adds additional variability which could make the distribution of the test statistic *Nr*^2 ^deviate from the -distribution. In order to study the effects of the additional variability on the distribution of the test statistic, we perform simulations under the null hypothesis of no LD. The empirical distribution is then compared to the -distribution. Specifically, we consider 2 bi-allelic SNPs, A and B. The alleles at marker A are denoted as *A*_1 _and *A*_2 _with frequencies *p*_1 _and *p*_2 _respectively and those at marker B are *B*_1 _and *B*_2 _with respective frequencies *q*_1 _and *q*_2_. When an individual is heterozygous at both markers, the underlying haplotypes cannot be identified with certainty from the genotype. In the first set of simulations, we assume the two markers are in Hardy-Weinberg equilibrium (HWE). Under HWE, the genotype frequencies at SNP A are , 2*p*_1_*p*_2_, and  for *A*_1_*A*_1_, *A*_1_*A*2 and *A*_2_*A*_2_, respectively. Similarly, we can write the genotype frequencies at SNP B. Under the null hypothesis of no LD, the joint distribution of the two-locus genotype follows a multinomial distribution with cell probabilities equal to the product of the corresponding genotype frequencies at the two SNPs because genotypes at the two SNPs are independent. For example, the two-marker genotype frequency of *A*_1_*A*_1_*B*_1_*B*_1 _is . We simulate the genotypes at the two SNPs in 1000 individuals by sampling from this multinomial distribution. The haplotype counts are then estimated from the simulated genotype data using the maximum-likelihood approach implemented in the R package "genetics". We then compute the test statistic *Nr*^2 ^based on the estimates of haplotype counts. We generate 10,000 replications for the simulation. The empirical distribution of *Nr*^2 ^from the 10,000 replicates is then compared with the -distribution. To examine the effect of ignoring the variation in haplotype estimation, we use the upper 0.05 quartile from the -distribution as cutoff and compute the proportion of simulated replicates with the test statistic exceeding the cutoff point. Similar analyses are performed at 0.01 and 0.001 significance level.

In our second set of simulations, we do not assume HWE. We denote the departure of genotype frequencies from HWE proportions as Hardy-Weinberg disequilibrium (HWD), which can bias estimates of haplotype frequencies for most likelihood-based methods [12]. We perform simulations under HWD to study its effect on the distribution of the test statistic, *Nr*^2^. Under HWD, the genotype frequencies at SNP A can be expressed in terms of allele frequencies, *p*_1_, *p*_2 _and a coefficient of HWD, *D_H _*,(3)

This represents a simple parameterization of genotype frequencies similar to those in [8] and [9]. Under HWD, there are less heterozygotes compared to the case under HWE when *D_H _*< 0, and more heterozygotes when *D_H _*> 0. Similar expressions can be written for the genotype frequencies at SNP B. Under the null hypothesis of no LD, the joint distribution of the two-marker genotypes is a multinomial distribution with each cell probability equal to the product of the corresponding genotype frequencies at the two SNPs. Two-marker genotypes for 1,000 individuals are simulated by sampling from this multinomial distribution. Similar to the case under HWE, haplotype counts are estimated using the maximum-likelihood approach implemented in the R package "genetics" and the values of the proposed test statistic *Nr*^2 ^are computed and compared with the -distribution.

### Our Approach

As shown in the results section, with unknown haplotypes, the empirical distribution of the test statistic *Nr*^2 ^deviates drastically from the -distribution, and type I error is greatly inflated. Therefore, we propose an Monte-Carlo approach for LD testing based on the distribution of the test statistic *Nr*^2 ^under the null hypothesis of no LD. We use the quartiles from the empirical distribution under the null hypothesis as the critical values in order to give the correct type I error rate. The distribution under null hypothesis is generated using a bootstrap approach [13]. Specifically, under the null hypothesis of no LD, the genotypes at the two SNPs are independent. Therefore, given the observed genotypes from a sample of *N *individuals, we first generate a bootstrap sample of *N *individuals by sampling with replacement from the genotypes at SNP A. This process is repeated for SNP B. The bootstrapped genotypes from SNP A are then randomly paired up with those from SNP B to form two-locus genotypes for the *N *individuals. This constitutes one bootstrap sample. We then apply the likelihood-based method in the R package "genetics" and calculate the test statistic for each bootstrap sample. This is replicated 10,000 times to generate the distribution under the null hypothesis.

### Power Analysis

We consider two simulation scenarios for the power analysis, one assuming HWE and the other under HWD, using the same simulation algorithm for both scenarios. The simulations are performed in two steps. First we simulate genotypes for 1,000 individuals at SNP A with HWD coefficient *D_H _*by sampling from the multinomial distribution with cell probabilities given in Equation (3). For the simulations under HWE, we set *D_H _*= 0. In simulation step two, for each of the two homologous chromosomes (one paternal and one maternal) in an individual, the allele at SNP B on the same chromosome is then sampled from a conditional distribution determined by the LD between SNP A and SNP B. From the definition of the statistic *D*, the haplotype frequencies can be expressed as

The conditional probability of the allele *B*_1 _at SNP B given that SNP A has allele *A*_1 _on the same chromosome is given by,

Similarly, we have *P*(*B*_2_|*A*_1_) = *q*_2 _- *D*/*p*_1_,*P*(*B*_1_|*A*_2_) = *q*_1 _- *D */*p*_2_, and *P*(*B*_2_|*A*_2_) = *q*_2 _+ *D*/*p*_2_. With the simulated genotypes, haplotype estimation and computation of the LD measure, *r*^2^, are performed using the R package "genetics". We carried out 10,000 simulation replications under this scenario, and the proportion of replications with *Nr*^2 ^greater than the cutoffs from the empirical distribution from the simulations under null hypothesis of no LD is taken as an estimate of empirical power. We compare our approach with two previous methods for testing LD, allowing for unknown haplotype, namely, the method by Weir [8] and the method based the asymptotic distribution [11]. We apply their tests to the same simulated samples to get the corresponding estimate of power. The cutoffs are based on the empirical distribution of the *Nr*^2 ^under the null hypothesis of *D*' = 0. We compute the empirical power for various values of *D*' ranging from 0 to 0.25 at significance level *α *= 0.05, 0.01 and 0.001.

## Results

### Simulation study

We perform simulations to study the effect of haplotype estimation on the distribution of the test statistic *Nr*^2 ^and compare it with the expected -distribution when haplotypes are known. For the two-biallelic SNPs in our model, only double heterozygotes have uncertain haplotypes. To maximize the effect of haplotype uncertainty, we set the *p*_1 _= *q*_1 _= 0.5 so that the double heterozygote frequency is maximized. We simulate 10,000 replications, each of which has 1,000 individuals, with genotypes at 2 SNPs under HWE and null hypothesis of no LD. The proportion of replications with test statistic greater than the quartiles from the -distribution is taken as an estimate of the empirical type-I error rate. Table 1 gives the empirical type-I error rates evaluated at several levels.

**Table 1:** Type-I error rate using  test with unknown haplotypes

	HWE		HWD	
		
Level	Type-I error	Inflation factor	Type-I error	Inflation factor
0.05	0.2859	5.72	0.3116	6.23
0.01	0.2252	22.52	0.2458	24.58
0.001	0.1662	166.2	0.1923	192.3

It is evident from Table 1 that the type-I error rate is inflated if we use the *χ*^2 ^test ignoring the uncertainty in haplotype estimation. At 0.05 level, type-I error rate is inflated by 5.72 times. It is inflated even further as the level of the test decreases. At 0.001 level, it is inflated by 166.2 times. This suggests that for samples with unknown haplotypes, the actual distribution of the test statistic differs drastically from the -distribution, especially under the tail, and therefore, using the usual *χ*^2 ^test will result in grossly erroneous conclusions.

Table 1 also gives the empirical type-I error rate under HWD. Similar to the case of HWE, type-I error rate is inflated. It is inflated further as the level of test decreases. At 0.05 level, it is inflated by 6.23 times and at 0.001 level, it is inflated by as much as 192.3 times. Compared to the result under HWE, type-I error rate is inflated further. This is probably due to the fact that HWD could bias the haplotype estimate. Therefore, our results suggest that the additional variability brought by the haplotype estimation makes the distribution of the test statistic differs drastically from the expected -distribution regardless of whether the SNPs are in HWE or not. Since we could generate the empirical distribution of the LD measure *r*^2 ^under the null hypothesis, a direct test of LD could be based the empirical distribution rather than relying on erroneous assumptions.

### Power Analysis

Power analyses are performed for the Monte Carlo-based tests based on 10,000 simulated samples, each containing 1,000 individuals under both HWE and HWD. Table 2 gives the power estimates for the simulations under HWE, at 3 significance levels, 0.001, 0.01 and 0.05.

**Table 2:** Power comparison of our test and two previous tests from simulations under HWE

	*α *= 0.05			*α *= 0.01			*α *= 0.001		
			
*D*'	our test	comp-LD	asym-LD	our test	comp-LD	asym-LD	our test	comp-LD	asym-LD
0	0.0500	0.0540	0.052	0.0100	0.0103	0.0105	0.0010	0.0011	0.0011
0.025	0.1232	0.1209	0.1201	0.0356	0.0324	0.0316	0.0059	0.0052	0.0048
0.05	0.3504	0.3468	0.3452	0.1564	0.1467	0.1455	0.0429	0.0386	0.0372
0.075	0.6696	0.6656	0.6643	0.4238	0.4095	0.4081	0.1810	0.1706	0.1659
0.1	0.8895	0.8847	0.8803	0.7200	0.7083	0.7051	0.4474	0.4325	0.4188
0.125	0.9814	0.9794	0.9726	0.9243	0.9218	0.9194	0.7598	0.7500	0.7259
0.15	0.9982	0.9979	0.9939	0.9875	0.9873	0.9869	0.9319	0.9301	0.9286
0.175	0.9998	0.9996	0.9992	0.9978	0.9975	0.9969	0.9875	0.9868	0.9856
0.2	1.0000	1.0000	1.0000	0.9999	0.9999	0.9997	0.9990	0.9988	0.9987
0.225	1.0000	1.0000	1.0000	1.0000	1.0000	1.0000	0.9998	0.9997	0.9995
0.25	1.0000	1.0000	1.0000	1.0000	1.0000	1.0000	1.0000	1.0000	1.0000

We change the level of LD by varying *D*' from 0 to 0.25. As shown in Table 2, the power of the Monte Carlo-based test increases quickly as *D*' increases. It reaches the perfect power of 1.0 at *D*' = 0.2 for *α *= 0.05 and *α *= 0.01. We apply the test based on composite LD to the same simulated data set for the purpose of power comparison. Table 2 also gives the power estimates for the test based on composite LD (labeled as "comp-LD" in the table) and the test based on asymptotic distribution (labeled as "asym-LD" in the table). It is obvious from Table 2 that the power of our test is comparable to the test based on composite LD, though our proposed method has a slight advantage. The test based on asymptotic distribution has the lowest power among the there tests.

Table 3 gives the power analysis results based on 10,000 simulated samples with 1,000 individuals each, under HWD. Similar to the results under HWE, the power of the Monte Carlo-based test increases quickly with increasing LD between the two SNPs. The power reaches 1.0 for *D*' = 0.15 at 0.05 level and for *D*' = 0.2 at 0.01 level. Our test also has comparable power with the test based on composite LD with our Monte Carlo-based test having a slight power advantage.

**Table 3:** Power comparison of our test and two previous tests from simulations under HWD

	*α *= 0.05			*α *= 0.01			*α *= 0.001		
			
*D*'	our test	comp-LD	asym-LD	our test	comp-LD	asym-LD	our test	comp-LD	asym-LD
0	0.0500	0.0505	0.0503	0.0100	0.0102	0.0103	0.0010	0.0013	0.0012
0.025	0.1538	0.1523	0.1519	0.0553	0.0516	0.0508	0.0122	0.0093	0.0088
0.05	0.4643	0.4642	0.4638	0.2480	0.2372	0.2366	0.0888	0.0752	0.0732
0.075	0.8006	0.8000	0.7897	0.5984	0.5858	0.5836	0.3388	0.3057	0.3021
0.1	0.9612	0.9610	0.9607	0.8838	0.8783	0.8779	0.7047	0.6728	0.6705
0.125	0.9958	0.9957	0.9952	0.9811	0.9799	0.9781	0.9267	0.9119	0.9106
0.15	0.9999	0.9999	9.9998	0.9987	0.9984	9.9981	0.9912	0.9899	0.9883
0.175	1.0000	1.0000	1.0000	1.0000	1.0000	1.0000	0.9998	0.9997	0.9995
0.2	1.0000	1.0000	1.0000	1.0000	1.0000	1.0000	1.0000	1.0000	1.0000
0.225	1.0000	1.0000	1.0000	1.0000	1.0000	1.0000	1.0000	1.0000	1.0000
0.25	1.0000	1.0000	1.0000	1.0000	1.0000	1.0000	1.0000	1.0000	1.0000

We have implemented the Monte Carlo-based test in R. The program can be downloaded from the author's website at http://www.biostat.mcg.edu/~hxu/software/ldtest.zip.

### Application

We apply our LD test to the SNP data from the genome-wide association study of the North American Rheumatoid Arthritis Consortium (NARAC). The NARAC sample consists of 868 Rheumatoid Arthritis cases and 1,194 healthy controls. The total data set contains 545,080 SNP-genotypes from the Illumina 550K chip. To illustrate the applicability of our test, we randomly choose 2 pairs of SNPs with different physical distance. The distance between rs3094315 and rs12562034 is 15.9 k basepairs and that between rs3094315 and rs11807848 is 308.7 k basepairs. The estimated *r*^2 ^and the p-value from our test are presented in Table 4. We perform the test in the cases and controls separately. It can be seen from the results in Table 4 that the LD patterns can be different in cases and controls.

**Table 4:** Application of our test to the NARAC data

SNP1	SNP2	distance (bp)	sample	*r*^2^	*P *- value
rs3094315	rs12562034	15882	cases	0.014	0.001
			control	0.016	0
rs3094315	rs11807848	308660	cases	0.00075	0.4424
			control	0.0045	0.0252

## Discussion

Testing the significance of LD between SNPs is of fundamental importance for genetic association studies. One popular measure of LD is *r*^2^. However, as shown in our simulations, for most population-based samples when the haplotypes are not known, the additional variability of haplotype estimation makes the traditional *χ*^2 ^test inapplicable. The departure form the assumed -distribution is more severe in extreme tails. This makes the *χ*^2 ^test even more problematic as extremely low significance levels are usually used to account for the effect of multiple testing in genome-wide studies. In this report, we propose a simple LD test based on the null distribution of the test statistic *Nr*^2 ^from simulations, taking advantage of the increasingly available computing powers. Unlike the test based on a composite LD measure, the Monte Carlo test is directly based on the distribution of the popular LD measure *r*^2 ^and can be report together with *r*^2^. As shown in the results section, our test has similar or slightly increased power compared to the test based on composite LD. The test is easily implemented in R. It works well with existing R packages and suitable for automation in large-scale genome-wide studies. A likelihood ratio test of LD using genotype data with unknown haplotypes has been developed by Slatkin *et al*. [14]. Similar to our approach, the null distribution of their test statistic is generated using computer-based permutations. However, the likelihood ratio test assumes HWE, while our test works well under either HWE or HWD. Nonetheless, similar to other permutation or bootstrap-based approach, the payoff of our approach is the computer running time, which is generally not a major concern as computing power increases.

Using simulations, we have considered the effect of haplotype estimation using the maximum-likelihood approach implemented in the R package "genetics" and showed that the additional variability brought by the haplotype estimation process cannot be safely ignored. This is an example of single imputation in statistics literature. Similarly, haplotype phase uncertainly can lead to problems in haplotype-phenotype association studies. In these studies, it is tempting to estimate haplotypes from genotype data using the existing haplotype estimation methods and assign the individuals with the most likely haplotype pair (or the pair with the highest posterior probability if a Bayesian method is used). The assigned haplotype pairs are then treated as true haplotypes in downstream association analyses. This two-stage approach, though simple, can lead to erroneous inference about the haplotype-phenotype association. Simulation studies have shown that this approach can lead to substantial bias in the estimated genetic effects, poor coverage of confidence intervals, and significant inflation of type I error [15-17]. For further discussions, please see [18] and [19]. Several methods have been developed to account for the uncertainty in haplotype estimation in the haplotype-phenotype association setting, including the expectation-substitution method [20] and the likelihood-based approach [21-23]. The latter involves the calculation of the variance-covariance matrix of the estimates based on the observed information matrix and has been implemented in the haplo.glm() function in the R package "haplo.stats" [22] and the program "HAPSTAT" [18].

Besides the maximum-likelihood method examined in the study, there are other more sophisticated methods for haplotype estimation that utilized high-density marker information, e.g. [24]. In humans, one can also utilize the information from large international collaborative efforts such as HapMap [25] and 1000 Genome Projects [26] for better haplotype estimation. It should be noted that our test is not novel but based on standard re-sampling procedure. However, the general simulation framework can be used to study the effect of other haplotype estimation methods because this is a two-step procedure. In the first step, the sample genotypes are simulated under the null hypothesis of no LD. The samples are then analyzed in the second step for haplotype estimation and computation of the final test statistic. Notice that we can use whatever method for haplotype estimation that are applicable in the second step. Therefore, the general simulation framework is rather flexible and can easily be extended to study the effect of other haplotype estimation methods. For example, in our study, we considered the haplotypes at 2 bi-allelic loci. It is straightforward to extend it to the cases with multiple SNPs. In the first step, genotypes at multiple SNPs can be generated using the standard bootstrap approach. In the second step, haplotypes at multiple SNPs can then be estimated using haplotype estimation methods for high-density markers. This approach could potentially offer some advantages over the likelihood approach because it relies on the empirical distribution of the final test statistic rather than the normal distribution. Indeed, simulation studies have shown that the likelihood based approach has strong bias away from the null hypothesis when haplotype diversity is high [19].

## Conclusion

We develop and implement a test of LD for population data when the haplotypes are unknown. It is directly based on the empirical distribution of *r*^2^, the measure of LD, and uses a Monte-Carlo approach. The test is easy to use and provides an alternative way to testing for LD for SNP data. It also provides a framework to study the effects of other haplotype estimation approaches.

## Competing interests

The authors declare that they have no competing interests.

## Authors' contributions

HX developed and implemented the method, and performed simulations. All authors contributed to analyzing and interpreting the results, and to writing the manuscript. All authors read and approved the final manuscript.
